# Calcium decreases cell wall swelling in sweet cherry fruit

**DOI:** 10.1038/s41598-022-20266-9

**Published:** 2022-10-03

**Authors:** Christine Schumann, Andreas Winkler, Moritz Knoche

**Affiliations:** Institute of Horticultural Production Systems, Leibniz University Hanover, Herrenhäuser Straße 2, 30419 Hannover, Germany

**Keywords:** Developmental biology, Plant sciences

## Abstract

Swelling of epidermal cell walls decreases cell-to-cell adhesion and increases cracking susceptibility in sweet cherry. Ca is suggested to decrease cracking susceptibility by crosslinking of cell wall components and, possibly, by decreasing swelling. The objective is to test this hypothesis. The effect of Ca on swelling of anticlinal epidermal cell walls was quantified microscopically in vivo using excised skin sections and in vitro using extracted cell walls. After removal of turgor, cell wall thickness increased. Incubation in CaCl_2_ decreased cell wall thickness up to 3 mM CaCl_2_. At higher concentrations thickness remained constant. Decreased cell wall swelling in vivo also occurred with other salts of divalent and trivalent cations, but not with those of monovalent cations. Decreased swelling was due to the Ca cation, the anions had no effect. Ca also decreased swelling of cell walls that were already swollen. CaCl_2_ also decreased swelling of extracted cell walls in vitro. There was no effect on swelling pressure. The effect on swelling increased as the CaCl_2_ concentration increased. Chlorides of divalent and trivalent cations, but not those of monovalent cations decreased swelling in vitro. The decrease in swelling among the divalent cations was linearly related to the radius of the cation. The results indicate that Ca decreases cracking susceptibility by decreasing swelling.

## Introduction

Rain-induced fruit cracking is a serious problem for producers of sweet cherry fruit in all areas worldwide where rainfall occurs before and during the harvest season^1^. Even low percentages of cracked fruit render a harvest uneconomic. Meanwhile, the quality of the remaining macroscopically un-cracked fruit is seriously compromised due to microcracking of the cuticle as a result of exposure of the fruit skin to surface moisture^2^. As a consequence of this microcracking, postharvest fruit transpiration is increased, fruit firmness is decreased, shrivel is more likely and the incidence of fruit rots is markedly increased^3^. Moreover, fruit skins with cuticular microcracks allow unrestricted water uptake^3,4^.

Reliable remedies against rain cracking are limited. They include the cultivation of trees under rain covers or in tunnels^5,6^. Except for extreme cases, cracking is markedly reduced, but the capital costs of production are markedly increased. Foliar applications of Ca-salts are reported to reduce rain cracking at times^7^. However, this effect is not reliably reproducible. In many cases, foliar Ca applications have no effect at all. A variety of mechanisms for the putative role of Ca in reducing rain cracking have been proposed.

First, the effects of Ca have been attributed to decreased water uptake as a result of a decrease in the driving force for osmotic water uptake. However, based on the concentrations of the Ca salts used, the osmotic potential of sweet cherry juice and the lack of a significant fruit turgor, the decrease in osmotic driving force is negligibly low and unlikely to be detectable in the field^3^. For example, for a spray concentration of 34 mM CaCl_2_ (equiv. to 0.5% CaCl_2_•2H_2_0), the osmotic potential of the solution would be − 0.25 MPa. At a fruit water potential of 3 MPa, this would correspond only to a 8.3% decrease in driving force^7^. Hence, decreased water uptake due to this osmotic effect can be excluded as a factor.

Second, Ca is known to increase crosslinking of cell wall constituents^8–10^. This also occurs in the load-bearing fruit skin of sweet cherry fruit^11,12^. Consistent with this observation is a shift in the fracture mode from (a) fracture by separation of adjacent cells along their middle lamella to (b) fracture across the cellulosic cell wall. The most likely explanation for these observations is a decrease in cell-wall swelling that results in increased cell-to-cell adhesion^12^.

The interactions between Ca ions and cell wall components have been studied in great detail (for a review see^13^). Basic studies employed well-defined linear pectins in standardized systems (pH, concentration of Ca and other cations). These studies led to the ‘egg box model’ that is widely used to describe the interactions of the Ca ion and homogalacturonans in cross linking^14,15^. The effects of Ca on isolated cell walls were investigated before^16–19^. In these studies, the focus was on the role of Ca in cell-to-cell adhesion during the preharvest^20^ and postharvest periods, and with respect to fruit quality traits such as firmness^21–23^. We are aware of no reports on the effects of Ca on cell walls at the tissue level or at the organ level.

The objective of our study was to establish the effects of Ca salts on cell wall swelling in mature sweet cherry fruit. Since the bursting of cells is associated with rain cracking and also that sweet cherry juice is acidic (pH 3.6), we were particularly interested in potential interactions between the effects of Ca and of pH on cell wall swelling.

## Results

**Figure 1:**
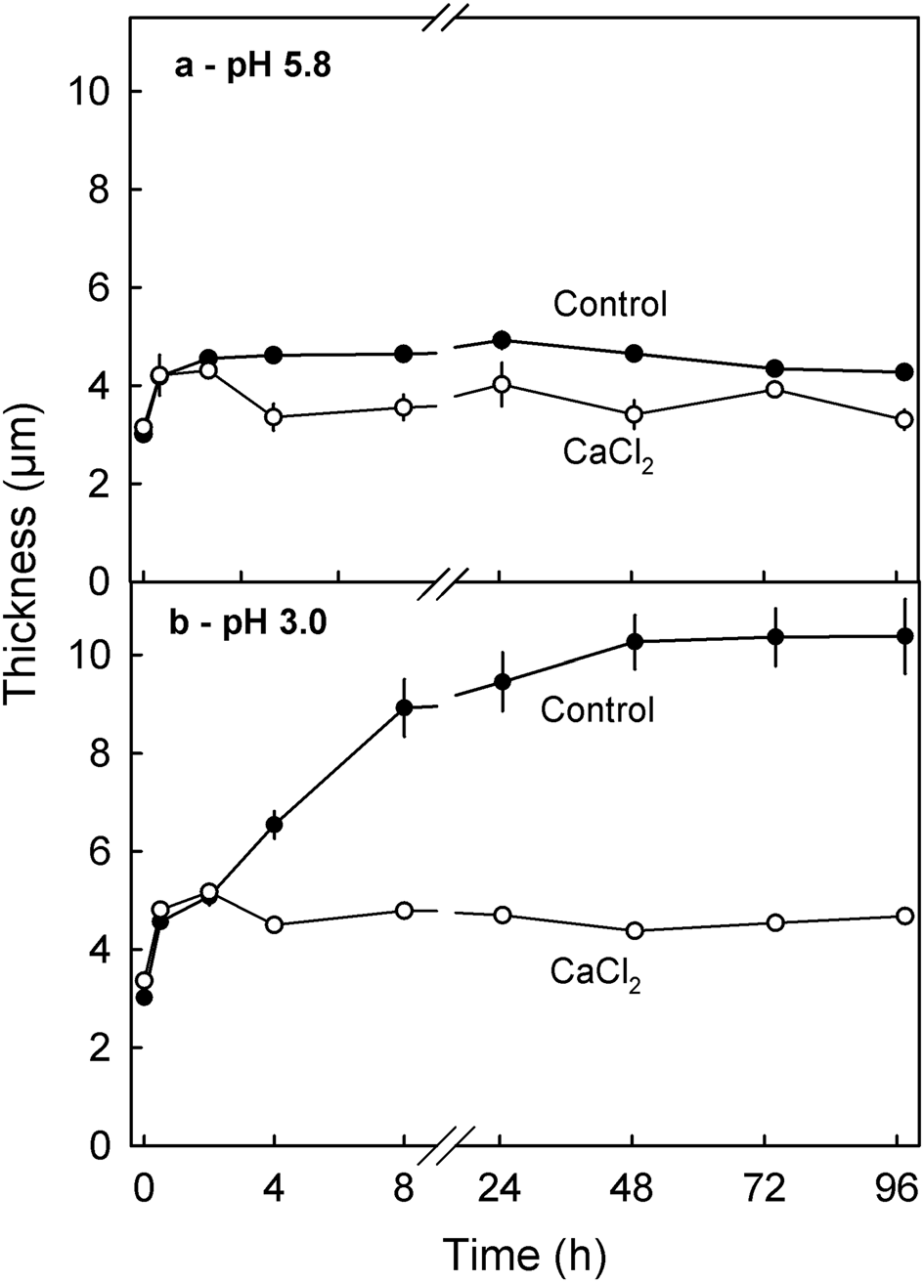
Time course of change in thickness of anticlinal cell walls of excised epidermal segments (ES) of mature ‘Santina’ sweet cherry fruit after a freeze/thaw cycle. The ES were incubated in buffered 10 mM CaCl_2_ or in buffer only. The concentration of the MES buffer was 10 mM MES and the pH was adjusted to pH 5.8 (**a**) or pH 3.0 (**b**).

Following release of turgor by a freeze/thaw cycle, cell wall thickness increased rapidly and reached an asymptote within 24 h when the pH was 5.8. The increase in cell wall thickness was larger at pH 3.0 than at pH 5.8. Incubation in CaCl_2_ resulted in less swelling. Decreasing the pH from pH 5.8 to pH 3.0 had little effect on swelling in the presence of CaCl_2_ as compared to the control without CaCl_2_ (Fig. 1).

**Figure 2:**
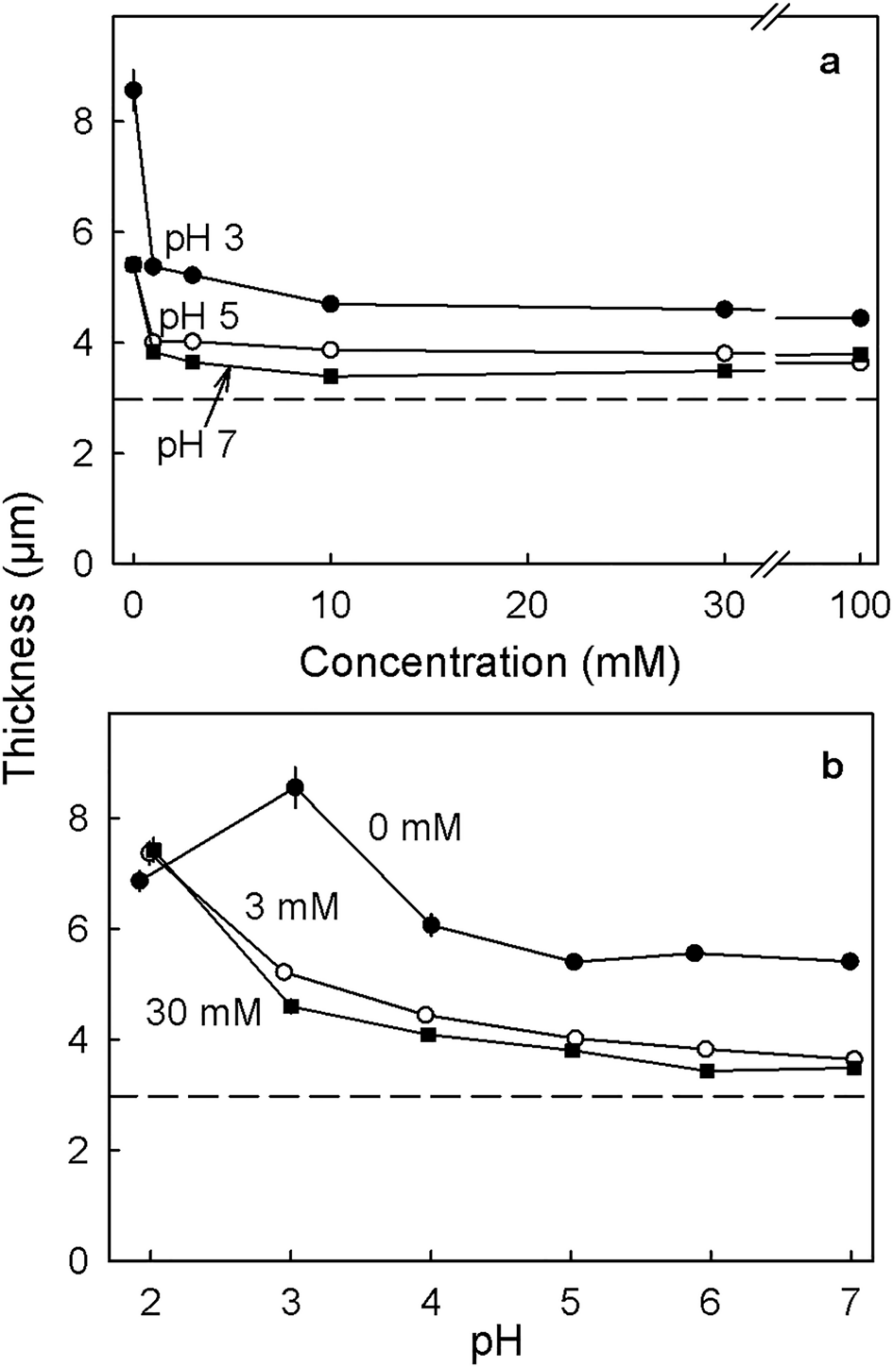
(**a**) Effect of the concentration of CaCl_2_ at pH 3.0, pH 5.0 or pH 7.0 on the thickness of the anticlinal cell walls of excised epidermal segments (ES) of mature ‘Burlat’ sweet cherry fruit. (**b**) Effect of pH at CaCl_2_ concentrations of 0, 3 and 30 mM. All ES were treated with a freeze/thaw cycle to release turgor followed by an incubation in the respective solution for 48 h. All solutions were buffered with 10 mM MES. The dashed horizontal line indicates the cell wall thickness in the native state, i.e., before the release of turgor.

At all pHs, CaCl_2_ decreased cell wall swelling after turgor release with the decrease depending on Ca concentration. There was little difference in response between concentrations of 3 mM and 100 mM (Fig. 2a). The decrease in thickness was largest at pH 3.0 and decreased as pH increased (Fig. 2b). The effect of CaCl_2_ on cell wall swelling was larger than that of increasing pH.

**Figure 3:**
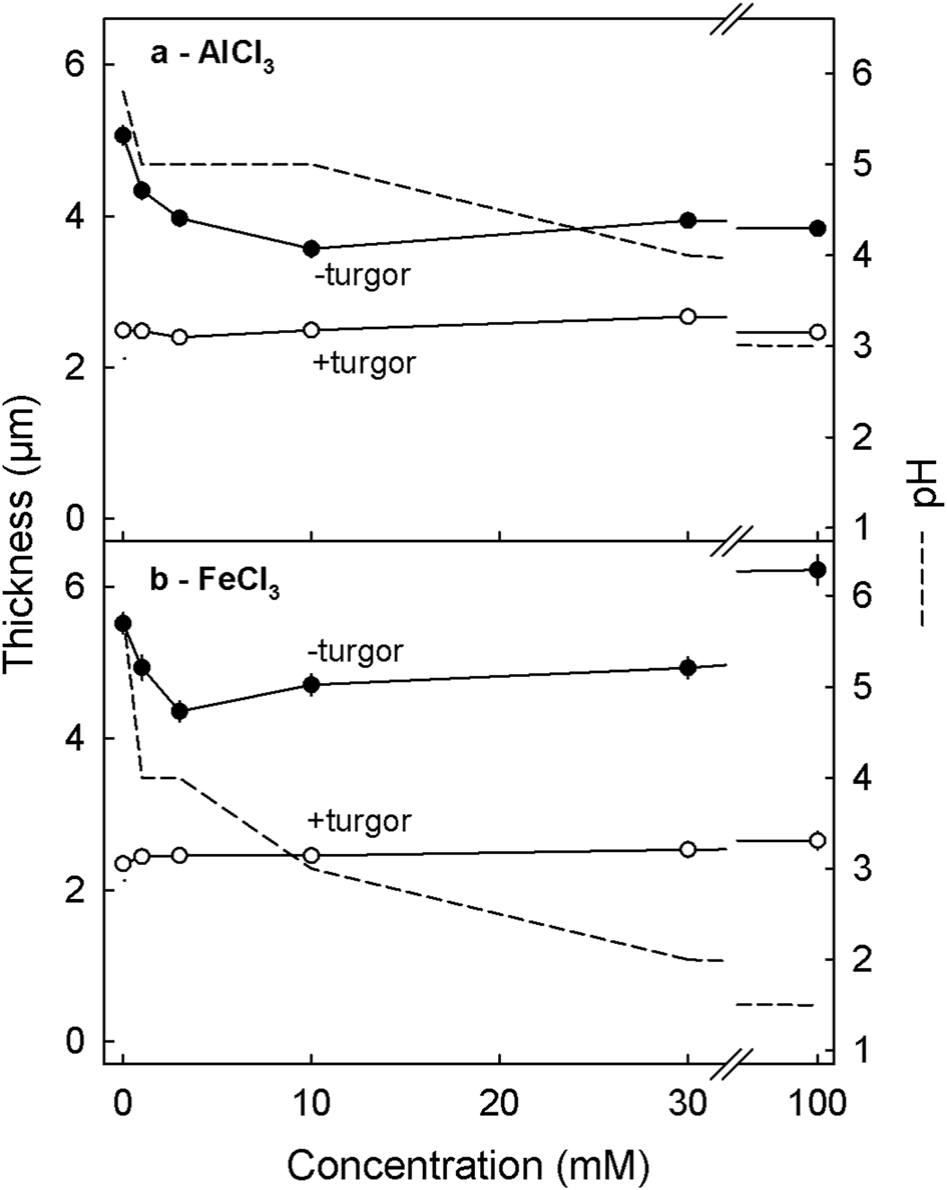
Effect of the concentration of AlCl_3_ (**a**) or FeCl_3_ (**b**) on the thickness of the anticlinal cell walls of excised epidermal segments (ES) of mature ‘Sam’ sweet cherry fruit. All ES were treated with a freeze/thaw cycle to release turgor followed by an incubation in one of a range of concentrations for 48 h. The dashed lines indicate the solution pH after incubation.

The trivalent cations AlCl_3_ and FeCl_3_ also decreased cell wall swelling in a concentration-dependent manner. These solutions, however, precipitated when buffered at higher pH. They were only stable at higher concentrations when the pH remained low. The decrease in cell wall thickness was largest for AlCl_3_ at 10 mM and for FeCl_3_ at 3 mM (Fig. 3a,b). It is interesting that 30 mM FeCl_3_ (pH 2.0) or 100 mM FeCl_3_ (pH 1.5) had no additional effect on cell wall swelling despite their very acid pHs. The decrease in swelling was not confined to CaCl_2_, AlCl_3_ and FeCl_3_ but also occurred with the other chloride salts of divalent cations. Only the chlorides of monovalent cations had no effect on cell wall swelling (Table 1).

**Table 1:** Effects of selected chloride salts on the thickness and swelling (Δthickness) of anticlinal epidermal cell walls of excised skin segments (ES) of ‘Sam’ sweet cherry fruit before (+ turgor) and after turgor release (−turgor) by a freeze/thaw cycle.

Treatment	Thickness (µm)		ΔThickness (µm)
	+ turgor	−turgor	
Control (MES)	2.9 ± 0.1	5.6 ± 0.1	2.7 ± 0.1
Control (water)	2.9 ± 0.1	5.6 ± 0.1	2.8 ± 0.1
KCl	2.8 ± 0.1	5.6 ± 0.1	2.8 ± 0.2
LiCl	2.9 ± 0.1	5.6 ± 0.1	2.7 ± 0.1
NaCl	2.9 ± 0.1	5.6 ± 0.1	2.7 ± 0.2
NH_4_Cl	2.9 ± 0.1	5.8 ± 0.1	2.9 ± 0.2
CaCl_2_	2.9 ± 0.1	3.7 ± 0.1*	0.9 ± 0.1
BaCl_2_	2.8 ± 0.1	3.6 ± 0.1*	0.8 ± 0.1
MgCl_2_	2.9 ± 0.0	4.6 ± 0.0*	1.7 ± 0.1
SrCl_2_	2.9 ± 0.1	3.9 ± 0.1*	1.0 ± 0.1

The ES were incubated in salt solutions at concentrations of 10 mM buffered with 10 mM MES. The pH was adjusted to pH 5.8 using KOH or HCl. Buffer only (10 mM MES, pH 5.8) and deionized water served as the controls. ΔThickness was calculated as the difference between cell wall thickness measured after turgor release, minus that measured before turgor release. Data are means ± SE.

*Mean separation within columns against the MES control indicated by *, Dunnett test at *p* < 0.05.

The decrease in cell wall swelling by CaCl_2_ was due to the Ca cation. There was no difference in cell wall swelling between various Ca salts, indicating the anion had essentially no effect on the response (Table 2).

**Table 2:** Effects of selected anions of Ca salts on thickness and swelling (Δthickness) of anticlinal epidermal cell walls of excised skin segments (ES) of ‘Sam’ sweet cherry fruit before (+ turgor) and after turgor release (−turgor) by a freeze/thaw cycle.

Treatment	Thickness (µm)		ΔThickness (µm)
	+ turgor	−turgor	
Control (MES)	2.8 ± 0.1	5.3 ± 0.1	2.5 ± 0.1
Control (water)	2.8 ± 0.1	5.2 ± 0.1	2.4 ± 0.1
CaCl_2_	2.8 ± 0.1	3.5 ± 0.1*	0.6 ± 0.1
Ca(NO_3_)_2_	2.8 ± 0.1	3.5 ± 0.1*	0.7 ± 0.1
CaSO_4_	2.8 ± 0.1	3.6 ± 0.1*	0.8 ± 0.1
Ca-acetate	2.9 ± 0.1	3.6 ± 0.1*	0.7 ± 0.1
Ca-propionate	2.8 ± 0.1	3.6 ± 0.1*	0.8 ± 0.1
Ca-formate	2.8 ± 0.1	3.6 ± 0.1*	0.8 ± 0.1
Ca-lactate	2.8 ± 0.1	3.6 ± 0.1*	0.8 ± 0.1
Ca-heptagluconate	2.8 ± 0.1	3.6 ± 0.1*	0.8 ± 0.1

The ES were incubated in salt solutions at concentrations of 10 mM and buffered with 10 mM MES. The pH was adjusted to pH 5.8 using KOH or HCl. Buffer only (10 mM MES, pH 5.8) and deionized water served as controls. ΔThickness was calculated as the difference between the thickness measured after turgor release minus that measured before turgor release. Data are means ± SE.

*Mean separation within columns against the MES control indicated by *, Dunnett test at *p* < 0.05.

Consistent with the effect of CaCl_2_ on cell wall swelling was the effect of EGTA. Incubating ES after turgor release in EGTA markedly increased cell wall swelling (+ 84%). The increase in swelling was slightly reduced when EGTA was applied together with CaCl_2_ (Table 3).

**Table 3:** Effects of CaCl_2_ (5 mM), EGTA (5 mM) or CaCl_2_ (2.5 mM) plus EGTA (2.5 mM) on thickness and swelling (Δthickness) of anticlinal epidermal cell walls of excised skin segments (ES) of ‘Burlat’ sweet cherry fruit before (+ turgor) and after turgor release (−turgor) by a freeze/thaw cycle.

Treatment	pH	Thickness (µm)		ΔThickness (µm)
		+ turgor	−turgor	
Control	5.8	2.3 ± 0.1 a^a^	4.9 ± 0.1 a	2.7 ± 0.2
Control	8.0	2.4 ± 0.1 a	4.8 ± 0.1 a	2.4 ± 0.2
CaCl_2_	8.0	2.1 ± 0.1 a	3.3 ± 0.1 b	1.2 ± 0.2
EGTA	8.1	2.2 ± 0.1 a	8.8 ± 0.3 c	6.6 ± 0.4
EGTA + CaCl_2_	8.0	2.3 ± 0.1 a	6.7 ± 0.3 d	4.5 ± 0.4

All solutions were buffered with 10 mM MES and pH adjusted with KOH to 8.0. MES at pH 5.8 and pH 8.0 served as controls. ΔThickness was calculated as the difference between the thickness measured after turgor release minus that measured before turgor release. Data are means ± SE.

^a^Mean separation within columns, Tukey’s Studentized range test at *p* < 0.05.

The decrease in cell wall swelling following incubation in CaCl_2_ was slightly reduced but remained significant, even after the CaCl_2_ solution was replaced by buffer only. Surprisingly, CaCl_2_ decreased swelling even of swollen cell walls (Table 4).

**Table 4:** Effects of CaCl_2_ on thickness of anticlinal epidermal cell walls of excised skin segments (ES) of ʻBurlatʼ sweet cherry fruit.

Treatment sequence	Thickness (µm)		
Phase I / phase II	Initial	Phase I	Phase II
−CaCl_2_/−CaCl_2_	3.2 ± 0.1 a^a^	6.5 ± 0.2 a	6.8 ± 0.2 a
−CaCl_2_/ + CaCl_2_	3.1 ± 0.1 a	6.3 ± 0.2 a	4.3 ± 0.1 b
+ CaCl_2_/−CaCl_2_	3.5 ± 0.1 b	3.9 ± 0.1 b	4.3 ± 0.1 b
+ CaCl_2_/ + CaCl_2_	3.2 ± 0.1 ab	3.9 ± 0.1 b	3.7 ± 0.1 c

The experiment was conducted by imposing sequential treatments on the same specimen. First, initial thickness of the cell walls was measured and then the turgor was released by a freeze/thaw cycle. The experiment then continued with two phases. The sequences of treatments (phase I / phase II) were −CaCl_2_/−CaCl_2_, −CaCl_2_/ + CaCl_2_, + CaCl_2_/−CaCl_2_, + CaCl_2_/ + CaCl_2_. The durations of phase I and phase II were both 48 h. At the ends of both phases, cell wall thickness was again measured. The concentrations of CaCl_2_ were all 10 mM. All solutions were buffered with 10 mM MES and pH adjusted with KOH to pH 5.8. Data are means ± SE.

^a^Mean separation within columns, Tukey’s Studentized range test at *p* < 0.05.

Incubating ES in hypotonic solutions (phase I) without CaCl_2_ had little effect on cell wall swelling. Under these conditions, cells remained turgid. However, when CaCl_2_, was added, cell wall thickness decreased also of turgid cells. This decrease was essentially independent of whether CaCl_2_ was added during phase I or phase II of the experiment. The decrease in cell wall thickness remained, even after CaCl_2_ was removed from the solution. Repeating the same experiment but incubating ES in hypertonic solutions during phase I, induced plasmolysis. The effect of CaCl_2_ was qualitatively the same as when incubating fruit first in hypotonic solutions. The only difference was that CaCl_2_ had a markedly larger effect on cell wall thickness in the absence of turgor. CaCl_2_ consistently reduced cell wall thickness even when cell walls were previously swollen (Table 5).

**Table 5:** Effects of CaCl_2_ and cell turgor on thickness of anticlinal epidermal cell walls of excised skin segments (ES) of ʻBurlatʼ sweet cherry fruit.

Treatment sequence	Thickness (µm)		
Phase I / Phase II	Initial	Phase I	Phase II
hypotonic − CaCl_2_ / hypertonic − CaCl_2_	2.8 ± 0.1 a^a^	3.1 ± 0.2 a	4.1 ± 0.1 a
hypotonic − CaCl_2_ / hypertonic + CaCl_2_	2.8 ± 0.1 a	3.2 ± 0.1 a	2.8 ± 0.1 bc
hypotonic + CaCl_2_ / hypertonic − CaCl_2_	2.7 ± 0.1 a	2.3 ± 0.0 b	2.8 ± 0.1 bc
hypotonic + CaCl_2_ / hypertonic + CaCl_2_	3.0 ± 0.1 a	2.3 ± 0.1 b	2.6 ± 0.1 c
hypertonic − CaCl_2_ / hypertonic − CaCl_2_	2.7 ± 0.1 a	4.8 ± 0.1 c	4.5 ± 0.1 d
hypertonic − CaCl_2_ / hypertonic + CaCl_2_	2.9 ± 0.1 a	4.6 ± 0.1 c	2.9 ± 0.1 bc
hypertonic + CaCl_2_ / hypertonic − CaCl_2_	2.9 ± 0.1 a	2.9 ± 0.1 a	3.3 ± 0.1 e
hypertonic + CaCl_2_ / hypertonic + CaCl_2_	2.9 ± 0.1 a	3.0 ± 0.1 a	3.2 ± 0.1 be

Cell turgor was manipulated by incubating ES in 0.25 M sucrose (hypotonic) or 1.5 M sucrose (hypertonic) in the presence of 10 mM CaCl_2_ (‘ + CaCl_2_’) or its absence (‘−CaCl_2_’). The experiment was conducted by imposing sequential treatments on the same specimens. First, initial thickness of cell walls was measured (‘initial’). The experiment then continued with two phases lasting 24 h each. All solutions were buffered using 10 mM MES at pH 5.8. At the end of each phase, cell wall thickness was measured. Data are means ± SE.

^a^Mean separation within columns, Tukey’s Studentized range test at *p* < 0.05.

Incubating ES in hypertonic solutions (phase I) resulted in plasmolysis and swelling of cell walls at pH 3.0, and less so at pH 5.8 (Table 6). When incubation continued and pH was increased from pH 3.0 (phase I) to pH 5.8 (phase II), cell wall swelling increased even more. There was no decrease in cell wall swelling, indicating the effect of pH on swelling was not reversible. Quantitatively similar data, albeit at a lower level, were obtained in plasmolyzed cells during incubation in a hypertonic solution at pH 5.8.

**Table 6:** Effects of pH and turgor on the thickness of anticlinal epidermal cell walls of excised skin segments (ES) of ʻAdriana’ sweet cherry fruit.

Treatment sequence	Thickness (µm)		
Phase I / phase II	Initial	Phase I	Phase II
hypotonic pH 3.0 / hypertonic pH 3.0	2.9 ± 0.1 ab^a^	9.5 ± 0.3 a	8.2 ± 0.3 a
hypotonic pH 3.0 / hypertonic pH 5.8	3.0 ± 0.1 ab	10.6 ± 0.4 b	11.9 ± 1.1 b
hypotonic pH 5.8 / hypertonic pH 3.0	2.8 ± 0.1 ab	3.2 ± 0.1 c	4.7 ± 0.1 c
hypotonic pH 5.8 / hypertonic pH 5.8	2.8 ± 0.1 ab	3.2 ± 0.1 c	4.0 ± 0.1 c
hypertonic pH 3.0 / hypertonic pH 3.0	2.7 ± 0.1 a	5.3 ± 0.1 d	5.7 ± 0.2 c
hypertonic pH 3.0 / hypertonic pH 5.8	2.8 ± 0.0 ab	5.6 ± 0.1 d	9.3 ± 0.3 a
hypertonic pH 5.8 / hypertonic pH 3.0	2.6 ± 0.1 a	4.0 ± 0.1 ce	4.8 ± 0.1 c
hypertonic pH 5.8 / hypertonic pH 5.8	2.7 ± 0.1 a	4.1 ± 0.1 e	4.0 ± 0.1 c

^a^ Mean separation within columns, Tukey’s Studentized range test at *p* < 0.05.

Turgor was manipulated by incubating ES in 0.25 M sucrose (‘hypotonic’) or 1.5 M sucrose (‘hypertonic’) at pH 3.0 or pH 5.8. The experiment was conducted by imposing sequential treatments on the same specimens. First, initial thickness of cell walls was measured (‘initial’). The experiment then continued with two phases (phase I / phase II) lasting 24 h each. All solutions were buffered using 10 mM MES. At the end of each phase, cell wall thickness was again measured. Data are means ± SE.

When intact fruit were pretreated by incubation in isotonic CaCl_2_, sucrose, EGTA or MES, cell wall thickness of CaCl_2_-treated skins remained low and not significantly different from the un-incubated controls whereas cell wall thickness increased in isotonic sucrose, EGTA and MES (Table 7). When turgor was released by a freeze/thaw cycle, cell wall thickness of CaCl_2_-treated fruit increased slightly but remained significantly lower than that of control fruit or of fruit pretreated with isotonic sucrose, EGTA and MES.

**Table 7:** Thickness of anticlinal cell walls of excised skin segments (ES) of ʻBurlatʼ sweet cherry fruit after pretreating whole fruits by incubation for 24 h in isotonic CaCl_2_ solution containing 10 mM MES, isotonic sucrose solutions without CaCl_2_ or in hypotonic 5 mM EGTA containing 10 mM MES or 10 mM MES buffer only.

Treatment	pH	Thickness (µm)		ΔThickness (µm)
		+ turgor	−turgor	
CaCl_2_	8.2	2.6 ± 0.1 a^a^	3.8 ± 0.1 a	1.3 ± 0.1
Sucrose	8.2	3.6 ± 0.1 bc	4.6 ± 0.1 b	1.0 ± 0.2
EGTA	8.2	3.8 ± 0.2 b	5.1 ± 0.1 c	1.3 ± 0.2
MES	8.2	3.4 ± 0.1 c	5.0 ± 0.1 bc	1.6 ± 0.2
Control	n.d	2.8 ± 0.1 a	4.9 ± 0.1 bc	2.1 ± 0.2

Thickness of cell walls was determined on ES after excision and pretreating intact fruit for 24 h (+ turgor). The turgor was then released by a freeze/thaw cycle (−turgor). After 48 h, the cell wall thickness was again measured. Fruits for control were held for 24 h at room temperature. Swelling (Δthickness) was calculated as the difference in cell wall thickness without turgor, minus that with turgor. Data are means ± SE.

n.d. not determined.

^a^Mean separation within columns, Tukey’s Studentized range test at *p* < 0.05.

**Figure 4:**
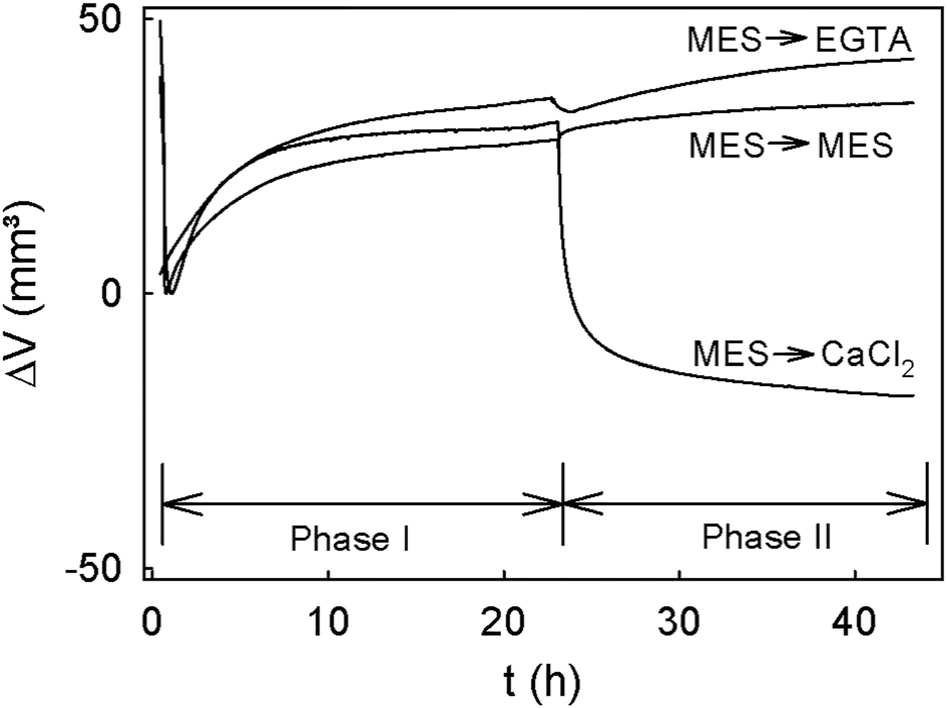
Time course of swelling of cell walls extracted from mature ‘Burlat’ sweet cherry. The experiment was conducted as a two-phase experiment by performing sequential treatments. During phase I, the cell walls were swelling in MES buffer. During the subsequent phase II, the buffer was replaced and swelling was monitored in buffered 10 mM CaCl_2_ or buffered 5 mM EGTA (phase II) or MES buffer only (10 mM). The pH of all solutions was adjusted to pH of 5.8. Swelling was quantified as the change in volume (∆V) of the cell wall at a constant pressure of 3.9 kPa.

The time course of change in volume of extracted cell walls during incubation in buffer revealed a transient decrease in volume that was followed by an increase in volume. The increase in volume reached an equilibrium within about 24 h (Fig. 4). When the MES buffer was replaced by buffered EGTA, cell wall volume increased slightly indicating increased swelling compared to the MES control. However, when the MES was replaced by buffered CaCl_2_, cell wall volume decreased markedly.

**Figure 5:**
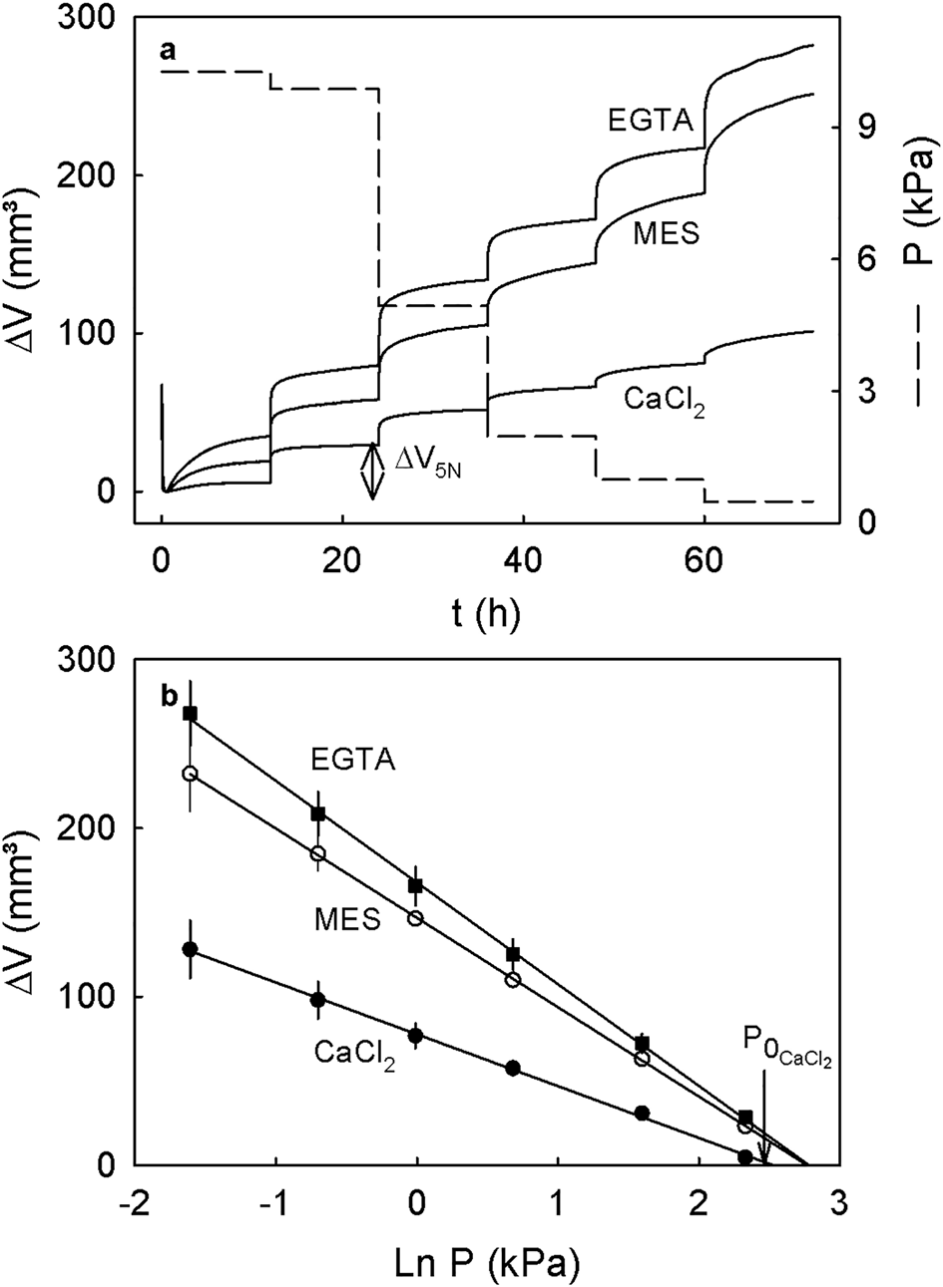
Effect of a stepwise decrease in pressure on swelling of extracted cell walls of mature ‘Burlat’ sweet cherry fruit. Cell walls were allowed to swell in the presence of buffered 10 mM CaCl_2_, buffered 5 mM EGTA or buffer only. The buffer was 10 mM MES at a pH of 5.8. Swelling was quantified in vitro as the change in volume (∆V) of the extracted cell walls as the pressure applied to the cell walls was decreased. (**a**) Time course of change in pressure and change on volume of cell walls as the pressure was decreased stepwise from 10.3 to 0.1 kPa. At each pressure step, the pressure was held constant for 12 h to allow equilibration of cell wall swelling. (**b**) Relationship between the swelling of cell walls (∆V) at equilibrium and the natural logarithm of the applied pressure. The swelling pressure P_0_ corresponds to the pressure at which no swelling occurs. The value P_0_ was estimated as the x-axis intercept of a regression fitted through a plot of ∆V vs. ln P. The change in cell wall volume per unit pressure represents the slope of this regression and may be interpreted as a volumetric modulus of elasticity of the cell wall.

Stepwise decreases in the pressure applied to the extracted cell wall material resulted in stepwise increases in volume. These increases were lowest in the presence of CaCl_2_ and highest in presence of EGTA (Fig. 5a). The relationship between the change in volume and the logarithm of the pressure applied was linear and highly significant (Fig. 5b). There were no significant effects of CaCl_2_ or EGTA on the swelling pressure. The slope of the relationship, however, was significantly decreased by CaCl_2_ (Table 8).

**Table 8:** Effects of CaCl_2_ (10 mM) and EGTA (5 mM) on in vitro swelling pressure (P_0_), the change in cell wall volume per unit pressure (ΔV * ln P^-1^) and the maximum volume after swelling at 0.2 kPa (ΔV_max_) of cell walls of mature ‘Burlat’ sweet cherry fruit.

Treatment	P_0_ (kPa)	ΔV * ln P^-1^ (mm^3^ * kPa^-1^)	ΔV_max_ (mm^3^)
Control (MES)	16 ± 3 a^a^	− 53 ± 6 b	232 ± 22
CaCl_2_	12 ± 1 a	− 30 ± 5 a	124 ± 18
EGTA	16 ± 1 a	− 60 ± 4 b	268 ± 19

Cell walls were extracted as the alcohol insoluble residue (AIR). All solutions were buffered using 10 mM MES. Buffer only served as control. The swelling pressure was calculated as the extrapolated x-axis intercept of a linear regression fitted through a plot of the change in AIR volume vs. the natural logarithm of the applied pressure (ln P). The change in AIR volume per unit pressure represents the slope of this regression and may be interpreted as the volumetric modulus of elasticity of the AIR. The pH of each solution was adjusted to pH 5.8 using KOH or HCl. Data are means ± SE.

^a^Mean separation within columns, Tukey’s Studentized range test at *p* < 0.05.

**Figure 6:**
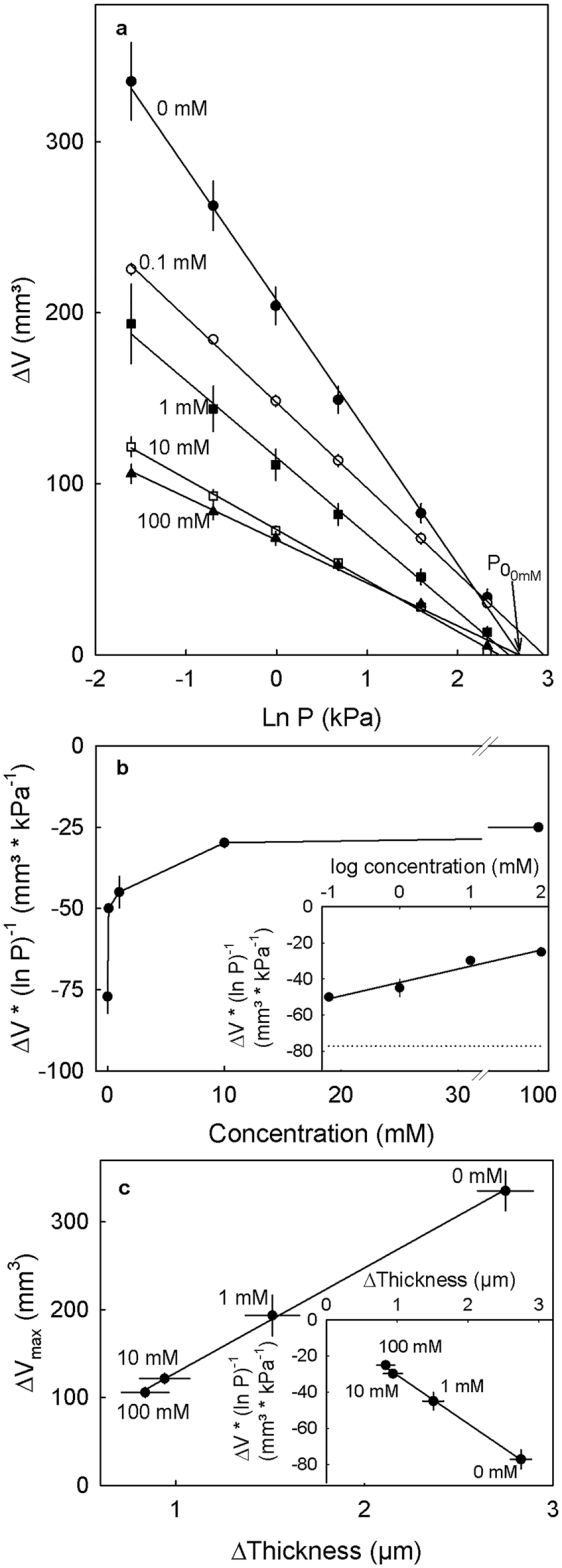
(**a**) Swelling of cell wall material extracted as the alcohol insoluble residue from ‘Burlat’ sweet cherry fruit when incubated in different concentration of CaCl_2_. All solutions were buffered with 10 mM MES and pH adjusted with KOH to 5.8. Swelling was quantified in vitro as the change in volume (∆V) at different pressures (P) using a custom-built pressure chamber. The ∆V of the swollen cell walls after loading the cell wall with different pressures was quantified. (**a**) Relationship between the swelling of cell walls (∆V) at equilibrium and the natural logarithm of the applied pressure. The swelling pressure P_0_ corresponds to the pressure at which no swelling occurs. The value P_0_ was estimated as the x-axis intercept of a regression fitted through a plot of ∆V vs. ln P. Each curve represents the mean of three repetitions. (**b**) Slope of the regression of ∆V vs. ln P depending on the CaCl_2_ concentration of the incubation solution. Inset: Slope ∆V vs. ln P depending on the logarithm of the applied concentration of CaCl_2_. The dotted horizontal line represents the control of 0 mM CaCl_2_. (**c**) Relationship between maximum swelling of the extracted cell walls incubated with different CaCl_2_ concentrations and the cell wall swelling examined on anticlinal cell walls of excised epidermal segments of ‘Burlat’ sweet cherry fruit incubated in the same incubation solutions. The ES were treated with a freeze/thaw cycle to release turgor followed by an incubation of 48 h. The cell wall swelling was calculated by the difference in the cell wall thickness after release of turgor minus that before release of turgor. Inset: Relationship of the slope ∆V vs. ln P and the microscopically determined cell wall swelling.

When the pressure applied to extracted cell walls was decreased stepwise in the presence of CaCl_2_, the corresponding increases in cell wall volume depended on both CaCl_2_ concentration and on the applied pressure (Fig. 6a). There was no consistent effect of CaCl_2_ concentration on swelling pressure (Fig. 6a). However, the slope of the regression lines did decrease significantly as CaCl_2_ concentration increased (Fig. 6b). This relationship was log linear and highly significant.

The effect of the Ca concentration on the swelling of extracted cell wall material mirrored that on cell walls as assessed by microscopy. Both, the slope (ΔV * ln P^-1^) and the ΔV_max_ were significantly related to the microscopically determined swelling (Fig. 6c).

**Figure 7:**
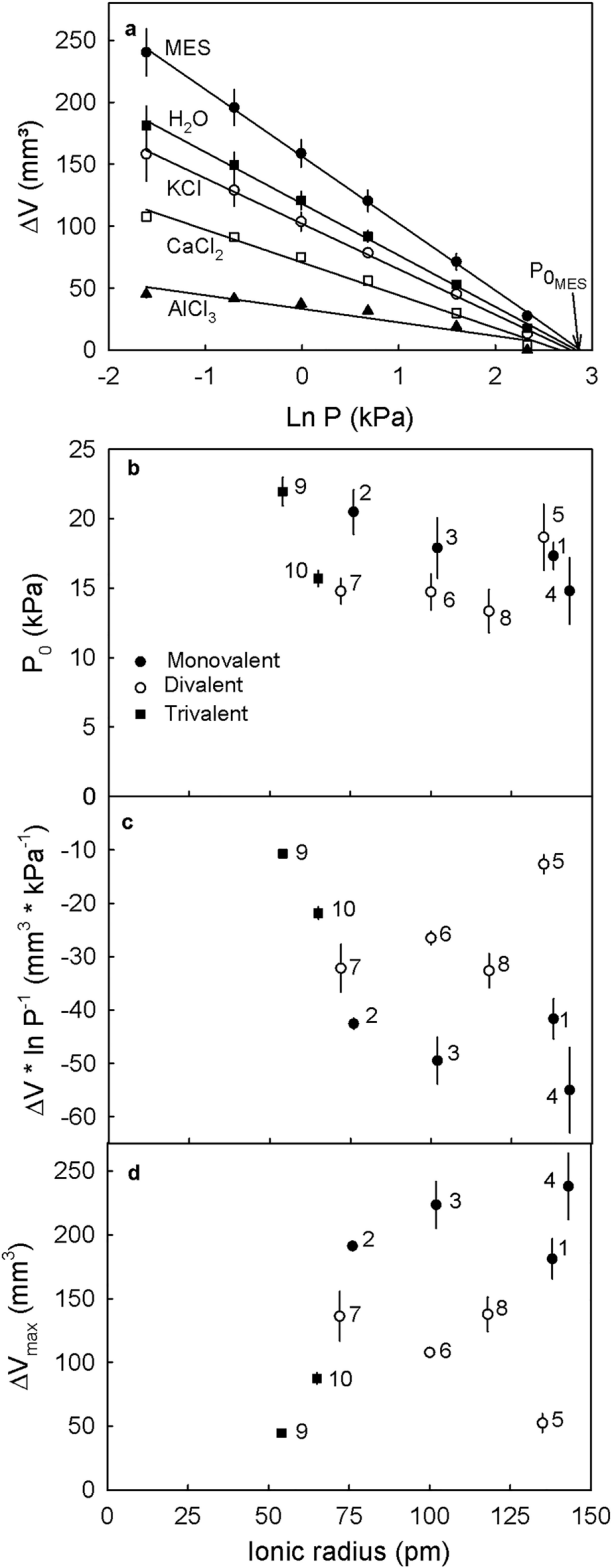
Swelling of cell wall material extracted from ripe ‘Burlat’ sweet cherry fruit when incubated in representative solutions of mono-, di- and trivalent cations. Solutions with monovalent and divalent cations were buffered with 10 mM MES and pH adjusted with KOH or HCl to pH 5.8. Solutions with trivalent cations were unbuffered and pH was measured (Table 9). 10 mM MES and deionized water served as control. Swelling was quantified in vitro as the change in volume (∆V) at different pressures (P) using a custom-built pressure chamber. The ∆V of the swollen cell walls after loading the cell wall with different pressures was quantified. (**a**) Relationship between the swelling of cell walls (∆V) at equilibrium and the natural logarithm of the applied pressure. The swelling pressure P_0_ corresponds to the pressure at which no swelling occurs. The value P_0_ was estimated as the x-axis intercept of a regression fitted through a plot of ∆V vs. ln P. Each curve represents the mean of three repetitions. (**b**) Swelling pressure P_0_ of solutions with different cations depending on the ionic radius of the cation. (**c**) Slope ∆V vs. ln P and maximum swelling of extracted cell walls (**d**) depending on the ionic radius of the cation in the incubation solution. (**b**–**d**) The numbers next to the data symbols refer to the following cations: KCl (1), LiCl (2), NaCl (3), NH_4_Cl (4), BaCl_2_ (5), CaCl_2_ (6), MgCl_2_ (7), SrCl_2_ (8), AlCl_3_ (9) and FeCl_3_ (10).

Cations that decreased the swelling of intact cell walls in vivo also decreased the swelling of extracted cell walls in vitro. Generally, there was no effect on P_0_. However, the slopes of plots of the relationship change of cell wall volume vs. the natural logarithm of the applied pressure increased markedly (less negative) in the presence of divalent and trivalent cations. Monovalent cations had no effect on P_0_, the slope term or the maximum volume (ΔV_max_) as compared to MES only. The effect of the divalent cations would seem to depend on the radius of the cation. The slope term (ΔV * ln P^-1^) and ΔV_max_ were both linearly related to ionic radius. The two trivalent cations must be excluded from this comparison. Their effects were confounded by the effects of pH. Here, solutions were un-buffered and highly acidic because neither solution was stable at a higher pH (Fig. 7 and Table 9).

**Table 9:** Effect of various chloride salts on the in vitro swelling pressure (P_0_), the change in cell wall volume per unit pressure (ΔV * ln P^−1^) and the maximum volume after swelling at 0.2 kPa (ΔV_max_) of cell walls of mature `Burlat` sweet cherry fruit.

Treatment	pH	P_0_ (kPa)	ΔV * ln P^-1^ (mm^3^ * kPa^-1^)	ΔV_max_ (mm^3^)
Control (MES)	5.7	18 ± 1	− 54 ± 4	240 ± 19
KCl	5.8	17 ± 1	− 42 ± 4	181 ± 16*
LiCl	5.8	21 ± 2	− 43 ± 1	191 ± 2
NaCl	5.8	18 ± 2	− 49 ± 4	224 ± 18
NH_4_Cl	5.8	15 ± 2	− 55 ± 8	238 ± 26
BaCl_2_	5.8	19 ± 2	− 13 ± 2*	53 ± 7*
CaCl_2_	5.8	15 ± 1	− 27 ± 1*	108 ± 4*
MgCl_2_	5.8	15 ± 1	− 32 ± 4*	136 ± 19*
SrCl_2_	5.8	13 ± 2	− 33 ± 3*	138 ± 13*
Control (water)	5.5	19 ± 3	− 34 ± 5	150 ± 17
AlCl_3_	3.8	22 ± 1	− 11 ± 1*	45 ± 2*
FeCl_3_	2.4	16 ± 1	− 22 ± 1*	88 ± 4*

Cell walls were extracted as the alcohol insoluble residue (AIR). All solutions were buffered using 10 mM MES and the pH adjusted to pH 5.8 using KOH or HCl. The only exceptions were AlCl_3_ and FeCl_3_ that were used without buffer. Buffer only served as control. The swelling pressure was calculated as the extrapolated x-axis intercept of a linear regression fitted through a plot of the change in AIR volume vs. the natural logarithm of the applied pressure (ln P). The change in AIR volume per unit pressure represents the slope of this regression and may be interpreted as a volumetric modulus of elasticity of the AIR. Data are means ± SE.

*Mean comparisons within columns against the MES control by Dunnett test, *p* < 0.05. Only AlCl_3_ and FeCl_3_ were used un-buffered and hence, are compared with the water control by Dunnett test, *p* < 0.05.

**Figure 8:**
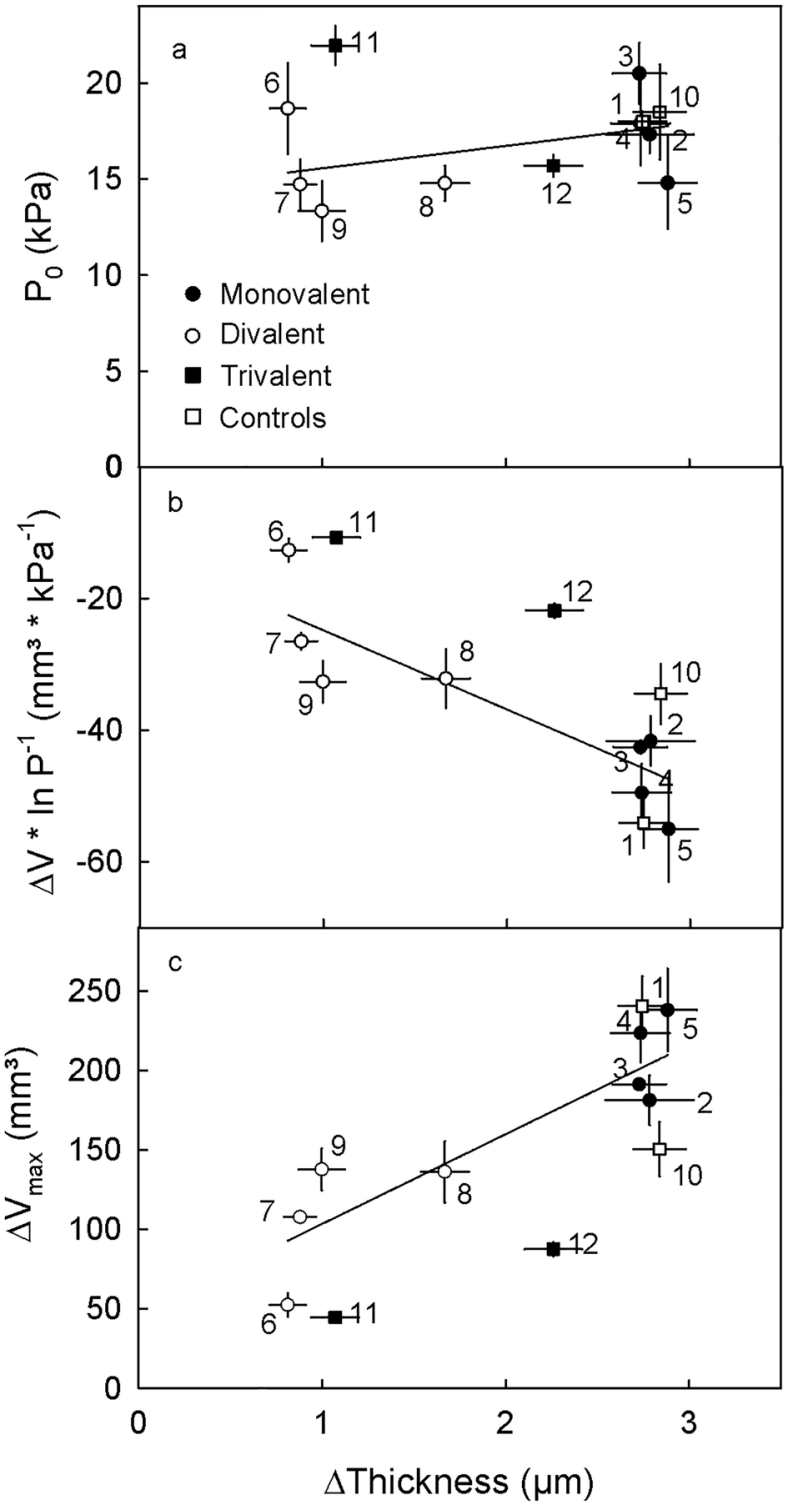
Relationship between the swelling pressure (P_0_) (**a**), the change in volume per unit pressure (ΔV * ln P^-1^) (**b**) and the maximum swelling volume (ΔV_max_) (**c**) of extracted cell walls in vitro and the swelling of cell walls in vivo as affected by mono-, di- and trivalent chlorides of the following salts: KCl (2), LiCl (3), NaCl (4), NH_4_Cl (5), BaCl_2_ (6), CaCl_2_ (7), MgCl_2_ (8), SrCl_2_ (9), AlCl_3_ (11) and FeCl_3_ (12). MES buffer (1) and water (10) served as controls. The mono- and divalent salts were buffered in 10 mM MES. Only the trivalent salts were not buffered. Swelling of extracted cell walls in vitro was determined using a custom-built pressure chamber, the swelling in vivo was determined by microscopy on skin sections.

There was no significant relationship between the swelling of the cell wall in vivo and the swelling pressure P_0_ of extracted cell walls in vitro (Fig. 8a). However, the slope term (ΔV * ln P^-1^) as well as the ΔV_max_ of extracted cell walls were both significantly related to the swelling of cell walls in vivo (Fig. 8b,c). Furthermore, these relationships did not differ from those established using a range of CaCl_2_ concentrations (Fig. 6).

## Discussion

Our discussion focuses on the following aspects: (1) The effects of calcium and other di- and trivalent cations on swelling of cell walls and (2) the effects of pH on the swelling of cell walls.

Our results indicate that divalent and trivalent cations decrease swelling of cell walls in vivo as determined by microscopy and also in vitro as determined by quantifying swelling pressure in extracted cell wall material. We infer that the decrease in swelling must have been due to a cross linking of negative charges in the cell wall. First, only cations carrying two or more charges were effective. There was no effect of monovalent cations. Second, the anion that would bind to any positive charge had no effect. Third, the crosslinking effects of Ca^2+^ on pectins are well documented and usually described using the analogy of the egg box model^14^. In this model, Ca represents the egg that “cross links” to adjacent chains of homogalacturonans. Fourth, pectins contain galacturonic acid as the dominating monomers. The acid function carries a negative charge at physiological pHs and, hence, will bind cations. Lastly, the middle lamella is comprised of pectin^8^. The crosslinking by di- or trivalent cations decreases cell wall swelling. Decreased swelling, in turn, increases cell to cell adhesion, thereby strengthening the cell wall. This behaviour is consistent with the observation that cell separation during cracking occurs by separation of adjacent cells and exposure of the middle lamellae at the crack surface—as opposed to the fracture of cellulosic cell walls^12,24^. Our results in sweet cherry are also consistent with observations in other crops^25^. It has been reported that cell to cell adhesion depended on the presence of Ca^2+^ cations and the degree of esterification^25^.

Among the divalent cations, the effects on cell wall swelling increase in order of the increasing radius of the cation. This effect is consistent with both the in vivo assay, using microscopy and thin sections of fresh fruit and with the in vitro assay, using extracted cell wall material and the determination of swelling pressure.

Among the divalent cations, the maximum volume of the swollen cell wall material (ΔV_max_) and the amount of swelling per unit pressure (ΔV * ln P^-1^) both correlated with the increase in swelling of intact cell walls observed by microscopy. In contrast, the swelling pressure P_0_ was unaffected by any of the cations. This is not surprising, considering that P_0_ represents the pressure that must be overcome by the cell walls before swelling begins. Earlier studies established that the value of P_0_ is the same order of magnitude as cell turgor (21 kPa^26,27^) indicating that in turgid cells it is the turgor that prevents cell wall swelling (Grimm and Knoche^28^ and Table 5).

Among the cations investigated, Ca was found effective in decreasing swelling. Also, the Ca cation has been found beneficial for fruit quality^7^. The effect of Ca on cell wall swelling was rapid and reversible, as inferred from the response of swelling to treatment with EGTA. EGTA has a high affinity for Ca and, hence, it extracts Ca from the cell wall thereby increasing swelling. It is worth noting that Ca also decreased the swelling of cell walls that were already swollen. These properties make Ca an ideal candidate for manipulating cell wall swelling at the whole-fruit level in the orchard. However, for Ca to be effective requires that Ca can reach the appropriate site of action in the cell wall, at a sufficiently high concentration. Due to the progressive loss of xylem functionality in the developing sweet cherry fruit^29^, vascular delivery from tree to fruit is very low, so instead Ca must be applied to the fruit directly as a spray^30^.

Swelling of cell walls depended not only on the presence of Ca ions but also on the pH of the incubation solution. At low pH, carboxyl groups of sugar acids of pectins are non-dissociated. As a result, the binding capacity for cations decreases^18^. In addition, more and more Ca^2+^ is replaced by H^+^ due to competition for the same binding sites. The decreasing binding capacity and the replacement of Ca by H both decrease crosslinking of cell walls, increase cell wall swelling and thereby weaken cell-to-cell adhesion. The effect of low pH is thus somewhat comparable to that of EGTA. However, in addition to the effect of EGTA, the number of sorption sites also decreases at low pH due to decreased dissociation. To study the interaction of Ca and pH we used living ES in which enzymes are still active. It has been shown, that the degree and the pattern of esterification also depended on pH^20^. At low pH, the activity of pectinmethylesterases increases leading to a lower degree of esterification. This, in turn, would thus oppose the effect of pH on dissociation. It is therefore conceivable, that the number of binding sites for cations increased at low pH. This could increase the effect of Ca at low pH despite a decrease in binding sites due to the dissociation of the carboxylgroups. In contrast to the effect of pH on dissociation, the effect of deesterification would be irreversible. This effect will also occur in vivo for an intact fruit on the tree, when water uptake through microcracks causes localized bursting of cells of the outer flesh^31^. The cells of the outer flesh are the most susceptible because they are large, thin-walled and have a more negative osmotic potential than the epi- and hypodermal cells of the skin^28,31^. Sweet cherry juice is rich in malic acid^32^. Upon cell bursting, malic acid enters the apoplast and the apoplast pH decreases. Malic acid extracts and complexes with cell wall-bound Ca, thereby weakening the cell wall. Local swelling is induced. Ca rapidly binds to the binding sites before the pH of the apoplast decreases thereby decreasing cell wall swelling (Fig. 1). These considerations demonstrate that Ca and pH interact in their effects on cell wall swelling. The lower the pH of the incubation solution, the larger is the effect of Ca in decreasing cell wall swelling. However, if pH drops below the pKa of galacturonic acids (pKa 2.8–4.1)^33^, any non-methylesterified carboxyl groups exist in the undissociated form^34^. Then, due to the too low density of negative charges, binding sites are absent and crosslinking is prevented (Fig. 2b at pH 2.0).

In contrast to the effects of Ca, the effect of pH on swelling is irreversible. Cell walls that had swollen when exposed to low pH, remain swollen when transferred to a solution of high pH. Apparently, the increasing number of binding sites at the higher pH remain free without the binding by divalent or trivalent cations. This would be the case if Ca was extracted at low pH and removed from the cell wall.

It is important to note that the effects of the trivalent AlCl_3_ and FeCl_3_ on swelling are confounded by a simultaneous effect of pH. Both salts produce highly acidic solutions that have pH values around pH 2.0. At this pH, cell adhesion is essentially eliminated. At the same time, swelling is greatly reduced due to the crosslinking of cell wall constituents by Fe^3+^ or Al^3+^. This interpretation is consistent with earlier findings demonstrating decreased cracking as a result of decreased cell wall swelling^35^.

### Practical implications

The results obtained in this study demonstrate that salts of divalent and trivalent cations markedly reduce cell wall swelling, most likely by crosslinking the pectin middle lamellae. Decreased swelling maintains and improves cell-to-cell adhesion which is an important factor in the cracking process of sweet cherry. Among the salts tested, Ca salts are effective and have an acceptable ecotoxicological profile. However, their limitation is their consistently low ability to penetrate an intact cuticle^7,36,37^. To take advantage of their potential to decrease cracking susceptibility, the Ca must come into contact with the emerging cracks. This may possibly be achieved by application of Ca sprays during or immediately after rainfall^38^.

## Material and methods

### Plant material

Mature sweet cherry fruit (*Prunus avium* L.) of the cultivars Burlat, Sam and Regina were harvested at commercial maturity based on color and size from the Horticultural Research Station of the Leibniz University in Ruthe (lat. 52°14’N, long. 9°49’E). All trees were grafted on ‘Gisela 5’ rootstocks (*Prunus cerasus* x *P. canescens*) and cultivated in a greenhouse or under a rain shelter. Fresh fruits were used on the day of harvest or frozen and stored at − 20 °C until cell wall extraction. Additionally, off-season ‘Santina’ sweet cherries from Chile were purchased locally. All fruits selected for experiments were uniform in size and free from visual defects.

### Microscopy

Cell wall swelling was quantified microscopically using the procedure described earlier^26^. Briefly, thin rectangular epidermal skin sections (ES) were excised using a razor blade from the equatorial region of the cheek parallel to the fruit surface. An ES was excised, carefully blotted with soft tissue paper and transferred to a droplet of test solution on a microscope slide. The ES was then inspected by light microscopy (BX-60; Olympus, Hamburg, Germany). Calibrated digital images (camera: DP73; Olympus) were taken at × 40 magnification and the wall thickness of the anticlinal cell walls between pairs of turgid epidermal cells was measured by image analysis (cellSens 1.7.1; Olympus Soft Imaging Solutions, Muenster, Germany). This wall thickness measure actually represents the sum of two abutting cell walls, belonging to the two adjacent cells, plus the thickness of the interfacing middle lamella. This measure also represents the cell wall thickness of turgid cells—we will call this specimen ‘ + turgor’. Removal of turgor is a prerequisite for cell wall swelling^26^. To enable cell wall swelling, the same ESs were then subjected to a single freeze/thaw cycle. They were frozen in 3 ml of test solution at -20 °C for at least 12 h, then returned to room temperature and held for 24 h. This freeze thaw cycle eliminated cell turgor—we now call this specimen ‘−turgor’. Cell wall-thickness was re-quantified as described above—this time between two non-turgid cells. Cell wall swelling was then calculated as the difference in thickness, after (–turgor) minus before (+ turgor) the freeze–thaw treatment. Unless otherwise specified, one ES was cut per fruit from 10 fruit per treatment. Two micrographs were taken per ES and two cell walls were measured per micrograph. The total number of observations per treatment was thus 40.

### Experiments

The time course of cell wall swelling of anticlinal cell walls was studied in ES from ‘Santina’ fruit. The ES were incubated in 10 mM CaCl_2_ buffered (10 mM MES) either at pH 5.8 or pH 3.0. There were two controls with buffer only (no CaCl_2_) one at each of the pHs. The pH was adjusted using HCl (pH 3.0) or KOH (pH 5.8). The cell wall thickness was measured as described above immediately after excision (0 h). The ES were then frozen for at least 12 h, after which cell wall thickness was again quantified at 0.5, 2, 4, 8, 24, 48, 72 and 97 h after thawing.

Potential interactions between CaCl_2_ and pH on cell wall swelling were determined using ES of ‘Burlat’. The CaCl_2_ concentrations were 0, 1, 3, 10, 30 and 100 mM. All solutions were prepared in 10 mM MES buffer and the pH adjusted to pH 2, pH 3, pH 4, pH 5, pH 6 and pH 7, using HCl for the pHs < pH 4.2 and KOH for the others. The initial cell wall thickness was determined immediately after excision of ES from 20 fruit. A separate set of ES (n = 10) were excised, frozen, thawed and incubated for 48 h in the respective salt solutions to determine cell wall thickness in the absence of turgor. Cell wall thickness was determined as described above.

The effect of concentration of AlCl_3_ and FeCl_3_ on cell wall swelling was studied on ES excised from ‘Sam’. The concentrations were 0, 1, 3, 10, 30 and 100 mM. Due to the solubility characteristics of AlCl_3_ and FeCl_3_, the pHs of these solutions were not buffered but their pHs were determined after termination of incubation. For each solution, at each concentration, cell wall thickness was determined immediately after excision of the ES and again following a freeze/thaw cycle and a subsequent 48 h incubation period in the respective test solution.

The effects on cell wall swelling of the chlorides of a range of mono- and divalent cations were determined using ES excised from ‘Sam’. The chlorides were selected because of their high water-solubility. The following salts were compared at a concentration of 10 mM KCl, LiCl, NaCl, NH_4_Cl, CaCl_2_, BaCl_2_, MgCl_2_ and SrCl_2_. Solutions were buffered with 10 mM MES and pH adjusted with either KOH or HCl to pH 5.8. Deionized water and 10 mM MES served as control. Cell wall thickness before and after the removal of turgor was determined.

The effects of the anion accompanying the Ca cation was investigated using CaCl_2_, Ca(NO_3_)_2_, CaSO_4_, Ca-acetate, Ca-propionate, Ca-formate, Ca-lactate and Ca-heptagluconate, all at 10 mM. All solutions were buffered with 10 mM MES and the pH adjusted using either KOH or HCl to pH 5.8. Buffer solution (10 mM MES) served as control. Cell wall thickness was determined immediately after ES excision and after a freeze/thaw cycle followed by a 48 h incubation period in the respective salt solution.

The effect of extracting Ca^2+^ from the ES was investigated using a chelating agent. The ES were incubated in 5 mM EGTA, 5 mM CaCl_2_ or in 2.5 mM CaCl_2_ plus 2.5 mM EGTA. All solutions were buffered using 10 mM MES and pH was adjusted to pH 8.0 using KOH. Buffer only (10 mM MES), pH 8.0 or buffer only at pH 5.8 served as controls. Cell wall thickness was determined before and after removal of turgor as described above.

Whether the effect of calcium on cell wall swelling was reversible or not was investigated in a two-phase experiment. Initial cell wall thickness was determined in ES of ‘Burlat’. The turgor was then removed by a freeze/thaw cycle. In phase I of the subsequent experiment, the non-turgid ES were incubated for 48 h either in 10 mM CaCl_2_ buffered with 10 mM MES or in 10 mM MES only. Thereafter, cell wall thickness was re-measured. For the following phase II, the ES were split randomly into two groups. One group was again incubated for 48 h in 10 mM MES and the second group was incubated for 48 h in 10 mM CaCl_2_ prepared in 10 mM MES. After this, cell wall thickness was re-measured. A sequence of treatments in 10 mM MES (phase I) / 10 mM MES (phase II) served as controls.

Potential interactions between the effects of calcium and of turgor were studied in another 2-phase experiment using ES from ‘Burlat’. Initial cell wall thickness was determined immediately after excision as described above. Thereafter, the ES were incubated for 48 h in either a hypotonic (0.25 M sucrose) or a hypertonic (1.5 M sucrose) solution with and without 10 mM CaCl_2_ (phase I). After re-measuring cell wall thickness, each group was then divided randomly into two subgroups that were incubated in a hypertonic solution (1.5 M sucrose) with (subgroup 1) or without (subgroup 2) 10 mM CaCl_2_ (phase II). After 48 h, cell wall thickness was re-measured. All solutions were buffered in 10 mM MES (pH 5.8). A buffered (10 mM) hypotonic sucrose solution served as control.

Potential interactions between low and high pH and turgor on cell wall swelling were studied in another two-phase experiment. Initial cell wall thickness was determined. Thereafter, (phase I) the ES were incubated for 48 h in hypotonic (0.25 M sucrose) or hypertonic sucrose (1.5 M sucrose) at pH 3.0 or at pH 5.8. The pH was then (phase II) adjusted to pH 3.0 using HCl or to pH 5.8 using KOH. After re-measuring cell wall thickness, each group was divided into two subgroups. These were incubated in a hypertonic solution (1.5 M sucrose) at pH 3.0 (subgroup 1) or at pH 5.8 (subgroup 2). After 48 h, cell wall thickness was re-measured. All solutions were buffered in 10 mM MES (pH 5.8). A buffered (10 mM) hypotonic sucrose solution (pH 5.8) served as control.

The effect on cell wall swelling of pre-treating whole fruit by incubation in CaCl_2_ was studied in ‘Burlat’. Fruit (n = 25) were sealed at the stylar scar and at the pedicel/fruit junction using a fast-curing silicone rubber (SE 9186 Clear; Dow Corning Corp., Midland, USA). Sealed fruit were then incubated for 24 h in deionized water, isotonic CaCl_2_, isotonic sucrose or in 5 mM EGTA. All solutions were prepared in 10 mM MES. In this experiment, the pH was adjusted to pH 8.2 using KOH to maintain the EGTA in solution. For the controls, untreated fruit were placed under a wet paper towel for 24 h. The ES were then excised and cell wall thickness measured as above. Subsequently, turgor was released by a freeze/thaw cycle followed by 48 h incubation in 10 mM MES. Cell wall thickness was then remeasured.

### Preparation of cell wall material

Cell walls were extracted as the alcohol-insoluble residue (AIR) from ‘Burlat’ sweet cherry fruit^26,39^. A sample of ten fruit was pitted after thawing, transferred into 4 ml per g tissue of 80% (v/v) ice-cold ethanol and homogenized for 2 min using a kitchen blender (Zauberstab M 160 WH; ESGE, Mettlen, Switzerland). The slurry was boiled for 30 min, cooled to ambient temperature and then filtered through glass fiber filter paper (Whatman GF/C; Sigma-Aldrich, St. Louis, MO, USA). After washing with 95% (v/v) ethanol, the insoluble residue was extracted with 3 ml per g tissue of chloroform:methanol (1:1, v/v) for 15 min, filtered, washed again with chloroform:methanol (1:1, v/v) and filtered again. Finally, the residue was washed with acetone and dried overnight. The AIR was then weighed and ground with pestle and mortar.

### Swelling pressure of extracted cell walls

The swelling pressure of the AIR was quantified using the method described before^26^. Briefly, 25 mg of AIR was weighed into a custom pressure chamber (25.5 mm diam.). The AIR was wetted using 20 ml of 70% (v/v) ethanol and a negative pressure of 20 kPa for 10 min, followed by 30 min at atmospheric pressure to allow the suspension to settle. The chamber was then positioned under a universal testing machine (BXC-FR2.5TN; ZwickRoell GmbH & Co. KG, Ulm, Germany). A stainless-steel frit (diam. 25.4 mm, 1.57 mm thick, pore diam. 10 µm) attached to a 50 N force transducer (KAP-Z; ZwickRoell) was used to pressurize the wetted AIR at 3.9 kPa and the system allowed to equilibrate for 30 min. The experiment was conducted in two-phases, using repeated observations on the same specimen. During phase I, the supernatant was replaced by 15 ml of 10 mM MES. The pressure was held constant at 3.9 kPa for 22.5 h and the change in volume of the cell wall was monitored. The effects of CaCl_2_ and of EGTA on swelling were established in phase II by replacing the MES buffer by either 10 mM CaCl_2_ in 10 mM MES or by 5 mM EGTA in 10 mM MES solution. Buffer only (10 mM MES) served as control. The pressure was held constant for another 22.5 h. Swelling was recorded as an increase in height of the frit and, hence, an increase in volume of the cell wall material using the distance transducer of the universal testing machine. By multiplying the measured change in height of the frit by the known cross-sectional area of the chamber, the change in volume of the cell wall due to swelling was calculated. The pH of all solutions was adjusted to pH 5.8. The experiment was carried out in triplicate where one replicate corresponded to a separate extraction of AIR from ten fruit.

The effect of CaCl_2_ and EGTA on swelling pressure was studied using the same experimental setup as above in a repeated measures design. The AIR (25 mg) was wetted in 70% (v/v) ethanol supported by a negative pressure of 20 kPa for 10 min, followed by 30 min at atmospheric pressure to allow the suspension to settle. The cell walls were then pressurized at 10.3 kPa. After a 30 min equilibration period, the aqueous ethanol was replaced by 15 ml of 10 mM MES buffer as control, by 10 mM CaCl_2_ in 10 mM MES or by 5 mM EGTA in 10 mM MES. All solutions were at pH 5.8. First, the pressure was held constant at 10.3 kPa for 12 h. At this pressure, cell wall volume was minimum. Next, the pressure was reduced stepwise to 9.9, 4.9, 2.0, 1.0, 0.5 and 0.2 kPa. After each pressure step, the new (lower) pressure was held constant for 12 h, and the new increased volume due to cell wall swelling was recorded as described above. The amount of swelling (ΔV) at anyone pressure step was calculated by subtracting the minimum volume of the cell wall material (Vmin) at 10.3 kPa from the final cell wall volume at the end of each step. The 10.3 kPa pressure approximated the turgor of a sweet cherry fruit, and thus the pressure experienced by the cell wall *in vivo*^27^. The swelling pressure (P_0_) was estimated as the x-axis intercept of a linear regression through a plot of ΔV at equilibrium, at each pressure, vs. the natural logarithm of the applied pressure (ln P). The value of P_0_ represents the pressure required to suppress cell wall swelling. The slope of the regression (ΔV * ln P^-1^) represents the volume change of the extracted cell wall material per unit pressure change. We interpret this slope as the volumetric modulus of elasticity of the compressed cell wall material and will subsequently refer to it as such. The swelling pressures were determined with three replicates, where one replicate corresponds to an individual extraction of AIR from ten fruit.

The effect of the concentration of CaCl_2_ on the swelling pressure was investigated using the protocol described above. Following an initial wetting, supported by a negative pressure of 20 kPa for 10 min and a 30 min equilibration period in 70% (v/v) ethanol, the supernatant was replaced by 0, 0.1, 1, 10 or 100 mM CaCl_2_. All solutions were buffered using 10 mM MES and pH adjusted to pH 5.8. The pressure was decreased stepwise as described above and the P_0_ and the slope term (ΔV * ln P^-1^) determined as described above.

The effects of mono-, di- and trivalent cations on swelling pressure were determined using the protocol described above. Here, following wetting of the AIR by 70% v/v ethanol, supported by a negative pressure of 20 kPa for 10 min and a 30 min equilibration period, the supernatant was replaced by KCl, LiCl, NaCl, NH_4_Cl BaCl_2_, CaCl_2_, MgCl_2_, SrCl_2_, AlCl_3_ or FeCl_3_ all at a concentration of 10 mM. Solutions with mono- and divalent cations were buffered with 10 mM MES and the pH adjusted to pH 5.8. Due to the limited solubility of AlCl_3_ and FeCl_3_ at pH 5.8, these solutions remained unbuffered. Deionized water and 10 mM MES buffer served as control. The pressure was decreased stepwise as described above and the P_0_ and the slope term (ΔV * ln P^-1^) determined as described above.

### Statistics and data presentation

All experiments were performed in accordance with relevant institutional, national, and international guidelines and legislation. Data in the Tables and Figures represent arithmetic means and standard errors. Where not visible, error bars were smaller than data symbols. Only in Figs. 4 and 5a are individual replicates shown. Data were analyzed by regression analysis and analysis of variance. Mean separation was carried out using Tukey´s Studentized range test or Dunnett´s test (*P* < 0.05, package multcomp 1.3–1, procedure glht, R version 3.3.2; R Foundation for Statistical Computing, Vienna, Austria). Significance of coefficients of determination (r^2^) at *p* < 0.05 is indicated by *.

## Data Availability

The datasets generated during the current study are available from the corresponding author on reasonable request.
